# Letter to the Editor: Slovak Metrological Society

**DOI:** 10.6028/jres.096.039

**Published:** 1991

**Authors:** Josef Mandak

**Affiliations:** Slovak Metrological Society, Bratislava, CSFR — Czechoslovakia

I should like to bring to the attention of the many readers of the *Journal of Research of the National Institute of Standards and Technology* the establishment of the Slovak Metrological Society (SMS).

SMS came into being on October 16, 1990, as the result of a special founding Congress held in DT Zilina with 137 delegates from the whole of Slovakia in attendance. It brings together individuals involved in both the technical and legal aspects of metrology and its aim is to help in developing metrology and measurement in Slovakia. SMS functions under the direction of the Czechoslovak Metrological Institute (ČSMÚ), the center of Czechoslovak metrology. Together with the Federal Weights and Measures Office (FÚNM), state metrological centers, centers of the calibration service, and production plant metrological centers, it establishes the principal tasks of present-day metrology in the Czechoslovak Federal Republic (CSFR).

The Congress was opened by Dr.-Ing. Weidlich, who presented a paper concerning the aims and tasks of the Slovak Metrological Society. Next on the agenda was the presentation of papers with the titles: “Future Organization of Czechoslovak Metrology and Calibration Service,” “Legal Adjustment of Metrology After 1990 and Its Impact upon Users’ Organizations,” and “Metrology—a Tool for Achieving Quality in Production and Services.” These papers were read by Ing. Brezina, director of ČSMÚ and member of the commission IMEKO PC 14, and others.

Attention was principally paid to: looking for ways to cooperate with organizations such as WECC, EUROMET, CEN/CENELEC, and others, as well as how to participate in activities within the framework of ISO and IEC; the unfinished state of the law “On Metrology: Parts I–VI”; different models to achieve quality, how to choose among them, how to demonstrate and document quality as recommended by the 9000 series of ISO quality standards; problems associated with the complex system for educating metrologists in the CSFR; and others.

The papers presented at the Congress together with a listing of the Congress participants are contained in the Congress proceedings titled: Foundation Congress of the Slovak Metrological Society, DT Žilina, 1990.

The Congress developed the rules of the Slovak Metrological Society as well as its activities for the coming 3 years. The emphasis will be on the following: the possibility of bringing together individuals and groups that are active in the field of metrology and measurement, or which show interest in the results of metrology; diffusing knowledge of metrology; protecting, representing, and safeguarding the interests of metrologists; providing special counseling, consultations, and expert service; cooperating to secure the training of new metrologists; and developing and introducing advanced certificate systems and procedures, especially from the standpoint of their metrological aspects. In summary, the management principles and tasks of the SMS have been established; a committee of 15 members, with Dr.-Ing. Weidlich as its head and a control board of three members, has been elected.

The establishment of SMS will support the growth of metrology in Slovakia, especially as the national economy is transformed into a market economy. The ultimate goal is to incorporate the CSFR into the community of developed countries of Western Europe.

Those interested in cooperation with or further information about the SMS should write to the author at the following address:

Associate Professor Josef Mandak
Slovak Metrological Society,
Tr. L. Novomeského IV/487,
842 55 Bratislava,
Czechoslovakia

